# Percutaneous therapy of a mediastinal lymphangioma with fibrin glue: case report with clinical success after 4 years

**DOI:** 10.1186/s12893-018-0339-x

**Published:** 2018-01-24

**Authors:** Siyu Zhou, Siyuan Dong, Jiang Du

**Affiliations:** Department of Thoracic Surgery, First Hospital of China Medical University, NO.155 of north Nanjing street, Shenyang, Liaoning China

**Keywords:** Introthoracic lymphangioma, Percutaneous therapy, Fibrin glue

## Abstract

**Background:**

Lymphangioma of the mediastinum is a rare benign tumor, and most of the cases are treated by a surgical approach.

**Case presentation:**

This work reports the case of a 62-year-old female with a large lymphangioma extending from her neck to her abdomen with dysphagia, dyspnea, and cough for 2 months. Because of the location of the mass, only bilateral excision could remove the multiloculated cyst completely. However, the patient’s overall physical condition was very poor, and we thought she could not tolerate the bilateral surgery. Therefore, the patient was treated by percutaneous aspiration drainage followed by fibrin glue injection. Our method has never been reported in the treatment of such a large intrathoracic lesion thus far.

**Conclusions:**

Percutaneous puncture and aspiration drainage followed by fibrin glue injection may be a feasible treatment option for the therapy of intrathoracic lymphangioma, with less damage than with other therapies.

## Background

Lymphangioma is a benign tumor and considered a congenital malformation of the lymphatic system, which manifests as a dilated lymphatic vessel filled with lymph because of failure of the lymph vessel to connect to the venous system. Reports state that 47.5% to 90.0% of lymphangiomas occur in patients under the age of two years [1], and about 90% of the cases involve the axilla or neck, while less than 10% extend into the mediastinum [2]. About 1% of lymphangiomas are localized only to the chest, which is usually seen in adults.

In the current literature, fibrin glue has emerged as an effective substance for the therapy of neck and head lymphangiomas. This case report is of a 62-year-old female with a large lymphangioma extending from her neck to her abdomen that was successfully treated by percutaneous therapy which had never been reported.

## Case presentation

**Fig. 1:**
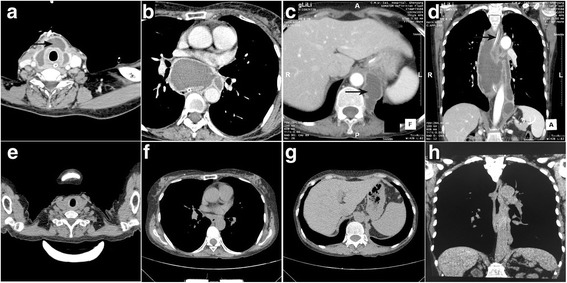
**a** Cervical level of the mass; **b** Aortic arch level of the mass; **c** Portal level of the mass; **d** The drainage tube(→) was inserted into the bottom of the mass; **e**-**h** 1.5 year after the operation

The study was approved by the Institutional Review Board of the first hospital of China Medical University. In 2013, a 62-year-old female presented to hospital with dysphagia, dyspnea, and cough for 2 months. The abovementioned conditions gradually increased, and she experienced fever for 10 days with a temperature of 38.5 °C. She had no personal or family history of illness. She had undergone cephalosporin sodium (Huabei, Hebei, China) treatment at the dose of 2 g twice a day for a week with no effect. Her white blood cell count was 12 × 10^9^/L. Physical examination revealed a neck mass. Computed tomography (CT) scan showed a large (54.1 × 16.2 × 7.2 cm), regular, and well-defined mediastinal cystic lesion extending from the neck into the abdomen with compression of the trachea and esophagus (Fig. 1a, b and c). Within the mass, there were multiple small cysts of variable size and revealed no enhancement after administration of contrast medium. Because of the location of the mass, only bilateral excision could remove the multiloculated cyst. However, the patient could not eat any food for a long time as the mass compressed the esophagus; her weight was only 37 Kg, and her overall physical condition was very poor. The patient was not a surgical candidate due to poor physical condition and was then referred for percutaneous treatment. A 10 French drain was placed inside the lesion from a cervical approach with the distal part of the drain placed at the inferior part of the lesion. (Fig. 1d). The drainage tube was placed for 20 days continuously with not positive suction and 1575 ml of milk white liquid was drained. The liquid was positive for lymph content and bacterial culture showed *Streptococcus viridans*. The diagnosis was thus made of an infected lymphangioma. Two days after therapy, the initial symptoms disappeared without complications. Twenty days after drainage, 10 ml commercially available kit of fibrin glue (porcine extraction, Beixiu, Guangzhou, China) were injected through the drainage tube to impel the lymphatic wall to adhere. The glue was composed of fibrinogen at a concentration of 30 mg/mL and 850 IU/mL of thrombin combined with tranexamic acid and calcium chloride. CT scan performed 1.5 years after treatment demonstrated complete resolution of the lymphangioma (Fig. 1e, f, g and h). We infer that the multiple small cysts might communicate with the biggest one, because after drainage, nearly all the small cysts were disappeared. This patient came to our hospital to review this year. At present the patient is remain asymptomatic. And she taken a CT at other hospital this year which manifested no recurrence (the same with the CT in this article). Regrettable, because the CT was not taken in our hospital, we cannot got a clear image which can be published in the article so we use the CT images she taken 1.5 year after treatment. When she come to our hospital next time we will try to get a clear CT in our hospital.

## Discussion and conclusion

Lymphangiomas are classified pathologically into three types: unilocular, cavernous, and intermediate. Regardless of the type, it is difficult to diagnosis before surgery. Additionally, mediastinal lymphangioma may remain asymptomatic for years and is usually found accidentally through physical examination. It can also be manifested with compression of intrathoracic structures, causing life-threatening complications, such as dysphagia and airway obstruction. The disease may also make the patient susceptible to secondary infection. This patient manifested both compression symptoms and infection. CT and magnetic resonance imaging (MRI) can demonstrate the extent of the lesion, growth pattern, morphology, and the relationship with intrathoracic structures. CT also shows calcification of the lesion better than MRI. However, MRI can reveal cystic characteristics and internal component of lesions completely [3]. In any case, the final diagnosis should be based on a combination of clinical, radiological, and histopathological findings.

At present, surgical excision remains the first-choice treatment. The prognosis is affirmative if the tumor is resected completely. The recurrence rate ranges from 0% to 27%, and the recurrence rate of partial resection is 50%. However, surgical treatment of mediastinal lymphangioma is difficult to achieve due to the extent of the lesion and the risk of damaging important organs. In this case, a 62-year-old patient in poor physical condition was deemed unsuitable for this surgery. We opted instead for draining the liquid completely first and injecting fibrin glue to impel the lymphatic wall to adhere. The result showed that this method not only relieved the symptoms but also provided a cure. A similar approach has been applied in the treatment of other diseases with smaller lesions, such as cysts [4, 5] and fistulas [6]. But this method had never been used in such a large intrathoracic mass. Moreover, in most cases the fibrin glue was used as sealant, whereas in this case, the fibrin gel was used as an adhesive.

There are also several types of adjunctive therapy such as sclerotherapy, for which 50% dextrose, bleomycin, Ethibloc, and OK-432 have been injected as sclerosing agents. Whereas, 50% dextrose has a high recurrence rate1. Bleomycin can lead to pulmonary fibrosis that is life-threatening. Dubois’ [7] study showed that use of Ethibloc as a sclerosing agent is an impactful and safe approach to cure the macrocystic and mixed type of lymphangioma. But, the case reported by Desir [8] manifested fever, chest pain and dyspnea for a month. OK-432 is also effective, but Shimizu [9] emphasized that with the injection of OK-432, temporary swelling and pyrexia may occur. They also pointed out that sclerotherapy may take a long time to produce the desired result and induce inflammation that may cause airway obstruction due to the transient enlargement of the lymphatic cavity.

Many reports advocate excision if possible when the lymphangioma is suspected. However, surgery was not suitable for this patient because of the size and extent of the lesion, which was from the neck to abdomen, and due to her poor overall physical condition. The patient’s condition was a contraindication for open thoracotomy. This study reports the successful percutaneous therapy of a large mediastinal lymphangioma, which was initially thought to be a palliative therapy but achieved a satisfactory outcome after 4 years. The drainage tube was placed for 20 days as a foreign body to fully drain the lesion to eliminate symptoms first and then cause an inflammatory reaction, causing the cyst wall to adhere. Then, a small dose of fibrin glue was used to promote and consolidate this procedure, and the cavity of the lymphangioma disappeared. As opposed to injecting a relatively large amount of sclerosing agents, this method worked more rapidly and the fusion of lymphatic wall and disappearance of lumen basically followed a natural physiological process without mechanical and chemical stimulation. Further, it may not lead to severe inflammation and airway obstruction. The original clinical manifestation gradually alleviated without any complications. After 4-year follow-up from discharge, the patient was healthy and had no recurrence.

Our method has never been reported in the treatment of such a large intrathoracic lesion thus far. It has obtained an unexpected but good result.

Percutaneous puncture and aspiration drainage may be a feasible treatment option, with less damage than with other therapies. This treatment is likely to be questioned in terms of prognosis and recurrence rate, but there is no doubt that this case offers an alternative reference to ease the pain of a patient who could not undergo the risk of surgery. This case report showed that percutaneous drainage of a large mediastinal lymphangioma followed by fibrin glue injection to seal the cavity allowed resolution of the patient’s symptoms with disappearance of the lesion and no clinical recurrence up to 4 years.

## Abbreviations

CT: Computed tomography
MRI: Magnetic resonance imaging

## References

[1] Hancock BJ, St-Vil D, Luks FI, Di Lorenzo M, Blanchard H (1992). Complications of lymphangiomas in children. J Pediatr Surg.

[2] Faul JL, Berry GJ, Colby TV, Ruoss SJ, Walter MB, Rosen GD, Raffin TA (2000). thoracic lymphangiomas, lymphangiectasis, lymphangiomatosis, and lymphatic dysplasia syndrome. Am J Respir Crit Care Med.

[3] Charruau L, Parrens M, Jougon J, Montaudon M, Blachere H, Latrabe V, Laurent F (2000). Mediastinal lymphangioma in adults: CT and MR imaging features. Eur Radiol.

[4] Jiang W, Qiu Q, Hao J, Zhang X, Shui W, Hu Z (2015). Percutaneous fibrin gel injection under C-arm fluoroscopy guidance: a new minimally invasive choice for symptomatic sacral perineural cysts. PLoS One.

[5] Murphy K, Oaklander AL, Elias G, Kathuria S, long DM (2016). treatment of 213 patients with symptomatic Tarlov cysts by CT-guided percutaneous injection of fibrin sealant. AJNR Am J Neuroradiol.

[6] Avalos-Gonzalez J, Portilla-deBuen E, Leal-Cortes CA, Orozco-Mosqueda A, Estrada-Aguilar Mdel C, Velazquez-Ramirez GA, Ambriz-Gonzalez G, Fuentes-Orozco C, Guzman-Gurrola AE, Gonzalez-Ojeda A (2010). Reduction of the closure time of postoperative enterocutaneous fistulas with fibrin sealant. World J Gastroenterol.

[7] Dubois J, Garel L, Abela A, Laberge L, Yazbeck S (1997). Lymphangiomas in children: percutaneous sclerotherapy with an alcoholic solution of zein. Radiology.

[8] Desir A, Ghaye B, Duysinx B, Dondelinger RF (2008). percutaneous sclerotherapy of a giant mediastinal lymphangioma. Eur Respir J.

[9] Shimizu J, Taga T, Kishimoto T, Ohta M, Tagawa K, Kunitsu T, Yamane T, Tsujita Y, Kubota Y, Eguchi Y (2016). Airway obstruction caused by rapid enlargement of cervical lymphangioma in a five-month-old boy. Clinical case reports.

